# The effect of orthodontic vertical control on the changes in the upper airway size and tongue and hyoid position in adult patients with hyperdivergent skeletal class II

**DOI:** 10.1186/s12903-022-02580-w

**Published:** 2022-11-24

**Authors:** Yining Liu, Wenyuanfeng Chen, Yu Wei, Guorui Zhang, Xinzhu Zhang, Hasan M. Sharhan, Baocheng Cao

**Affiliations:** School/Hospital of Stomatology Lanzhou University, No. 222, Tianshui South Road, Chengguan District, Lanzhou, 730000 Gansu Province China

**Keywords:** Hyperdivergent skeletal class II, Vertical control, Upper airway, CBCT analysis

## Abstract

**Background:**

At present, there are still controversies about the influence of orthodontic treatment on the size of upper airway and the position of hyoid bone. We investigated the effect of orthodontic vertical control therapy on the size of the upper airway and position of the tongue and hyoid bone in adult patients with hyperdivergent skeletal Class II.

**Methods:**

Overall, 15 adults with hyperdivergent skeletal Class II and normal occlusion, respectively, were selected as the experimental and control groups. The angle and line of the craniofacial structure, upper airway, hyoid bone position and three-dimensional (3D) upper airway indexes were measured using the Uceph 4.2.1 standard version and Mimics 21.0 software, respectively. The paired t-test, Wilcoxon symbol rank test, t-test of two independent samples, two independent sample nonparametric tests, Mann–Whitney U test, Pearson correlation analysis, the Univariate linear regression analysis and Multiple linear regression analysis were performed.

**Results:**

After treatment, the S-Go/N-Me (%) and the MP-SN and XiPm-SN angles were significantly different (*P* < 0.01). The U-MPW and PAS significantly increased (*P* < 0.05), sagittal diameter L_2_ increased significantly, and transverse diameter L_2_ decreased significantly (*P* < 0.01). Although no significant correlation was observed between the vertical change in the jaw and that in U-MPW and PAS, the sagittal diameter L_2_ showed a significant correlation (*P* < 0.05). The Multiple linear regression analysis showed that there was a significant negative correlation between the variables MP-SN and sagittal diameter L_2_ and positive correlation between S-Go/N-Me(%) and H-MP *(P* < 0.05). Furthermore, significant differences between the Hv (*P* < 0.01) and sagittal diameter L_1_(*P* < 0.05) were observed before and after treatment.

**Conclusions:**

After the orthodontic vertical control therapy in patients with hyperdivergent skeletal Class II, the upper airway only underwent adaptive changes during treatment without substantial size changes, the position of tongue body and hyoid bone did not change significantly. Furthermore, compared with normal occlusion, the velopharyngeal segment airway of patients with hyperdivergent skeletal Class II remains narrow and long after treatment.

**Supplementary Information:**

The online version contains supplementary material available at 10.1186/s12903-022-02580-w.

## Background

Since the development of orthodontics, airway complications have attracted the attention of orthodontists. Stenosis or collapse of the upper airway can cause obstructive sleep apnea-hypopnea syndrome (OSAHS), craniofacial deformities, arrhythmia, and increased blood pressure [1]. Moreover, hyperdivergent skeletal Class II malocclusion is a common clinical malocclusion. Brito et al. [2] showed that the volume size of the nasopharynx, velopharynx, and glossopharynx segments is basically the same in patients with different vertical skeletal types of skeletal Class II malocclusion. Additionally, Wang et al. [3] revealed that the smallest cross-sectional area of the nasopharyngeal segment and the volumes of the nasopharyngeal, velopharyngeal, and glossopharyngeal segments in the adult patients with hyperdivergent skeletal Class II group were smaller than those in the low-and normal-angle groups. Zhang et al. [4] treated adult patients with hyperdivergent skeletal Class II by extracting four premolars. They found no significant alterations in height, total height, and volume of each segment, total volume of the upper airway, or hyoid position. Although only the sagittal dimension of the oropharyngeal segment’s upper airway cross-sectional area was significantly reduced, the transverse dimension was increased considerably, and the cross-sectional area remained stable. Li et al. [5] extracted four premolars, after which they applied high J hook caps to strengthen anchorage, reverse Spee curve, and intermaxillary Class II traction to treat adult patients with hyperdivergent skeletal Class II. The result showed that the hyoid bone position shifted anteriorly and upwardly; however, the sagittal width of the upper airway did not change significantly. Shi et al. [6] also examined adult patients with skeletal Class II high angle. After extraction of the first premolars, the maxillary posterior teeth were intruded by the micro-implant anchorage (MIA) so that the mandible was counterclockwise. The minimum cross-sectional area of the oropharyngeal segment increased significantly compared to that before treatment, whereas the hyoid bone position remained unchanged. Consequently, it can be observed from the above that it may be affected by factors such as material sources, research methods, and treatment mechanisms. Moreover, whether adult patients with skeletal Class II high-angle can have upper airway abnormalities and whether they can affect the upper airway and hyoid bone position after orthodontic treatment remains controversial.

In this study, the upper airway size, airway height, minimum cross-sectional area, volume of each segment, total volume, and the hyoid bone position in adult patients with hyperdivergent skeletal Class II were examined through lateral cranial radiographs and CBCT (Cone beam Computer Tomography). Furthermore, the effect of orthodontic vertical control on the size of the upper airway and the tongue and hyoid bone positions in patients with hyperdivergent skeletal Class II was verified, which provided a foundation for clinical diagnosis and treatment.

## Methods

### Study participants

This is a retrospective study. The samples collected in this study depend on their availability. Overall, 15 patients who were admitted to the Orthodontics Department of Lanzhou University Stomatological Hospital from January 2019 to January 2022 and who fulfilled the inclusion criteria were selected, which included seven males and eight females (average age: 25.40 ± 3.28 years).

#### Inclusion criteria

Experimental group: 1) Permanent and complete dentitions; 2) Angle Class II, skeletal Class II, ANB angle > 4.7°, high angle (MP-SN ≥ 37.7°; S-Go/N-Me (%) ≤ 62%); 3) Using the straight arch-wire technology (MBT),14 cases (7 male and 7 female patients) and 1 case (female) were treated with extraction and non-extraction treatment, respectively. Furthermore, an individual normal standard was attained at the end of treatment. MP-SN angle, Y-axis angle, ANS-Me/N-Me (%), XiPm-SN angle decreased, or S-Go/N-Me (%) increased, and the surface type improved; 4) 18.5 kg/m^2^ ≥ body mass index (BMI) ≤ 24.9 kg/m^2^; and 5) Complete lateral cranial radiographs and CBCT images before and after treatment.

Control group: 1) Permanent and complete dentitions; 2) Angle Class I, skeletal Class I (0° < ANB ≤ 4.7°); 3) 18.5 kg/m^2^ ≥ BMI ≤ 24.9 kg/m^2^; and 4) Complete lateral cranial radiographs and CBCT imaging datas.

Exclusion criteria: 1) History of orthodontic treatment; 2) History of cleft lip and palate; 3) History of upper respiratory tract disease; 4) BMI ≥ 24.9 kg /m.^2^

### Measurement items and methods

The lateral cranial radiographs and CBCT images of all participants before and after treatment were obtained from the Department of Radiology, Lanzhou University Hospital of Stomatology. Lateral cephalogram was imported into Uceph 4.2.1 standard software (Intelligent Cephalometric Software,Sichuan,China) for the 2D measurement, and CBCT was imported into Mimics 21.0 software (Materialise’s interactive medical image control system, Leuven, Belgium) in DICOM form for the 3D measurement. Additionally, the same researcher measured all the data in this experiment. All measurement items were measured two times by the same researcher, the average value was taken, and the same researcher re-measured after 1 week. Kappa analysis was performed on the two measurement results. There was no significant difference between the two measurement results (κ = 0.688, *P* < 0.01).

**Fig. 1:**
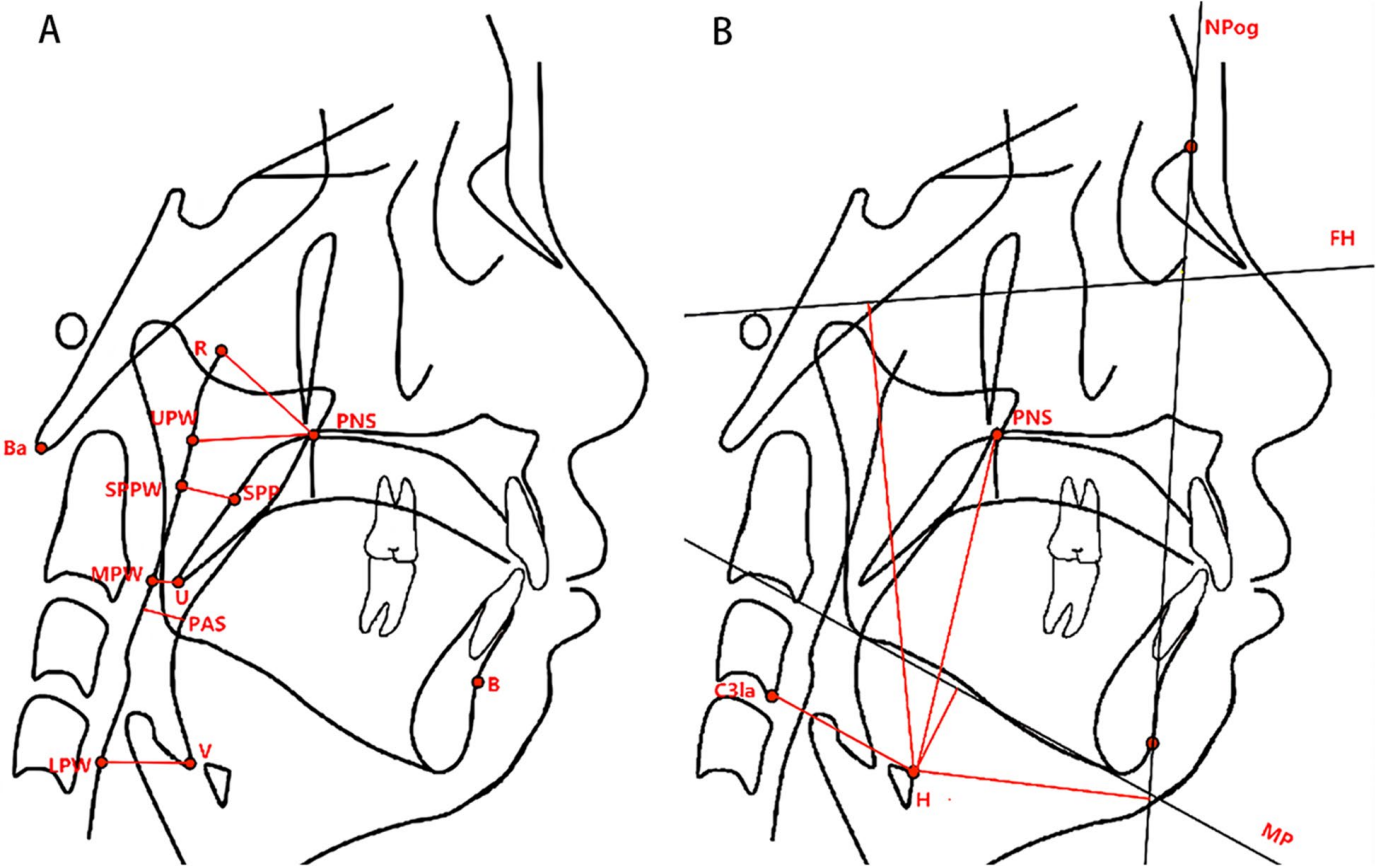
**A** Two-dimensional airway measurement indexes. **B** Hyoid bone position measurement indexes

The 2D measurement parameters included the following 12 items: Craniomaxilofacial structure angle and line distance parameters: SNA (Position of the maxilla relative to the skull),SNB (Position of the mandible relative to the skull), ANB (Positional relationship of the maxilla and mandible relative to the skull), MP-SN (The angle between the mandibular plane and the anterior skull base plane (SN), representing the inclination of the mandible), Y-axis (The anterior angle below the intersection of the center of the sella and the apex of the chin (SGn) and the plane of the eye and ear (FH), representing the chin constriction), ANS-Me/N-Me(%)(The height of the front and bottom/overall height means that 1/3 of the face and the bottom account for the proportion of the whole face.), S-Go/N-Me(%)(The rear height/overall height represents the proportion of the rear height in the whole face), XiPm-SN (Xi: a point in the center of the ramus. Pm: the intersection of concave-convex arcs in the frontal contour of the chin. XiPm: the corpus axis of the mandibular. XiPm-SN: Rotation of the mandibular body relative to the plane of SN [7]), U_1_- SN (The angle between the long axis of the maxillary central incisor and the plane of SN, representing the inclination of the maxillary central incisor), L_1_- MP (The angle between the long axis of the lower central incisor and the mandibular plane, representing the inclination of the lower central incisor), overjet (mm) (The horizontal distance that the maxillary teeth cover the mandibular teeth), and overbite (mm) (The vertical distance that the maxillary teeth cover the labial surfaces of the mandibular teeth). Upper airway sagittal parameters included the following six items: The distance between the PNS point (posterior nasal spine) and R point (R point: the posterior pharyngeal wall intersection with the PNS-Hor line (PNS-Hor line: Distance between PNS point and Hor point: the point located at the intersection between the greater wing and the body of the sphenoid bone): PNS-R (mm), the distance between the posterior nasal spine and the UPW point (the posterior pharyngeal wall intersection with the PNS-Ba line): PNS-UPW (mm), distance from SPP (from the center of the soft palate perpendicular to the intersection of the posterior pharyngeal wall and the posterior border of the soft palate) to SPPW (from the center of the soft palate to the intersection of the posterior pharyngeal wall): SPP-SPPW (mm), the distance between U (the apex of the soft palate) and MPW (the intersection point of the line perpendicular to the posterior pharyngeal wall through the U point): U-MPW (mm), the width of the airway along the line of Go-B: PAS (mm), the distance between V (base of the epiglottis) and LPW (the intersection of the line perpendicular to the posterior pharyngeal wall through the V point): V-LPW (mm).(Fig. 1A). Conversely, the hyoid bone position parameters included the following five items: vertical distance from H point (the uppermost and anterior point of the hyoid body) to the mandibular plane: H-MP (mm), that from the H point to the plane of FH: H-FH (mm), the distance from point H to C3la: H-C3la (mm), and the vertical distance between point H and NPog line: H-NPog (mm) (Fig. 1B).

**Fig. 2:**
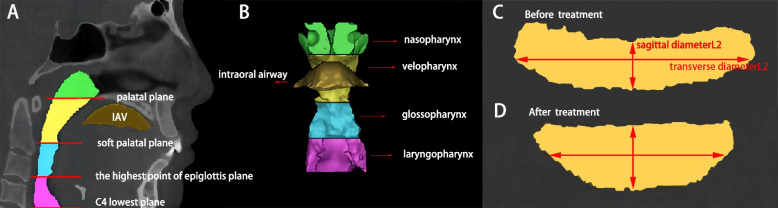
**A** Sagittal division of the upper airway; **B** 3D model of the upper airway and the intraoral space between the tongue and palate: green shown as the nasopharyngeal segment (the area from the roof of the airway to the plane of the posterior nasal spine); yellow shown as the velopharyngeal segment (the area from the plane of the posterior nasal spine to the plane passing through the apex of the soft palate); blue shown as the glossopharyngeal segment (the area from the plane of the apex of the soft palate to the plane passing through the apex of the epiglottis); pink is shown the laryngopharyngeal segment (the area from the apex of the epiglottis to the lowest plane of C4); brown is shown as the intraoral space between the tongue and palat; **C** Minimum cross-section of glossopharynx before treatment, the sagittal diameter L_2_ and transverse diameter L_2_ of the section; **D** Minimum cross-section of glossopharyngeal after treatment

The 3D measurement contents of the experimental group before and after treatment and the control group included the total airway volume (Vt), nasopharyngeal (Vn), velopharyngeal (Vv), glossopharyngeal (Vg), and laryngopharynx (Vl) sements volume (mm^3^), the height of the velopharyngeal and glossopharyngeal segments (Hv and Hg) (mm), the smallest cross-sectional area of the velopharyngeal and glossopharyngeal (S_1_ and S_2_) (mm^2^), minimum sagittal diameters (L_1_ and L_2_) (mm), transverse diameters (L_1_ and L_2_) (mm) of the velopharyngeal and glossopharyngeal airways of this section, and the intraoral space volume (IAV) between the tongue and palate (mm^3^) (Fig. 2A, B).

### Data analysis

SPSS 22.0 software (Statistical Package for Social Sciences,New York, USA) was used to analyze the normality of each measurement parameter. The paired t-test was performed on the measurement items before and after treatment to compare the changes when the measurement values attained a normal distribution pre-and post-treatment. However, when it did not follow a normal distribution, the Wilcoxon signed-rank test was performed before and after treatment on the measurement parameters to compare the changes. The Pearson’s correlation test was performed on the treatment results with significant changes to test the correlation between the different variables. The Univariate linear regression analysis was used to determine the independent variables and Multiple linear regression analysis was used to test the correlation between the independent variables of craniofacial structure and the dependent variables of upper airway, tongue body and hyoid bone position. The normality test was initially performed for the 3D airway measurement items in the experimental and control groups, the measurement group obeyed the normal distribution, the t-test for two independent samples was used to compare their differences. Moreover, when the measurement group did not follow the normal distribution, the independent sample nonparametric Mann–Whitney U test was used to compare the differences between the two groups. Statistical significance was considered set at *P* < 0.05.

## Results

**Fig. 3:**
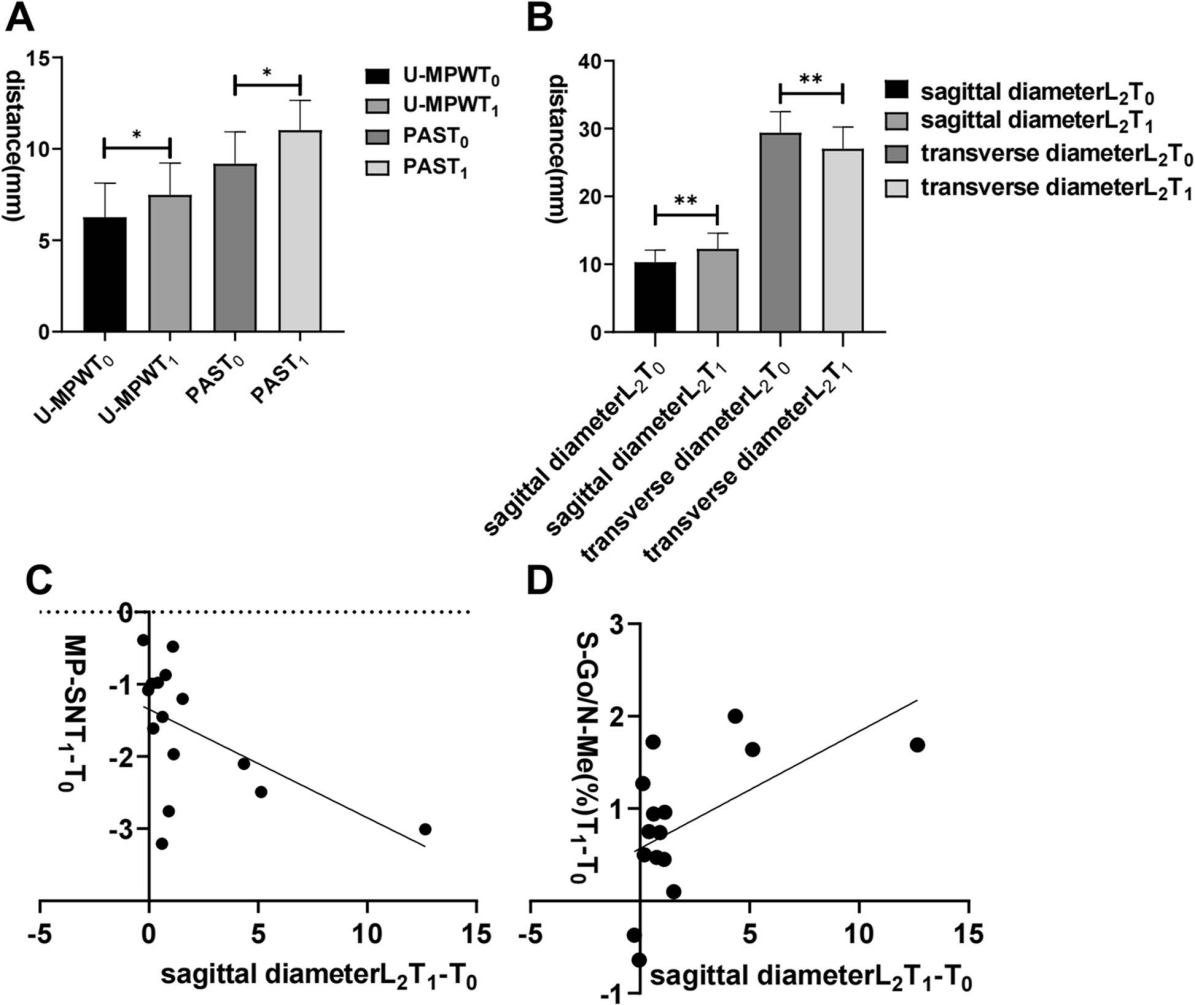
**A** Significant change metrics in the 2D direction of the upper airway. **B** Significant change metrics in the 3D direction of the upper airway. **C** Correlation between MP-SN change index and sagittal diameter L_2_ change index. **D** Correlation between S-Go/N-Me(%) change index and sagittal diameter L_2_ change index

Differences between the experimental and control groups were analyzed before treatment. A significant difference was not observed in the volume of each part of the upper airway, Vt, Hg, S_1_, S_2_, transverse L_1_, sagittal L_2_, transverse L_2_, or IAV. In addition, there were significant differences in Hv(*P* < 0.01) and sagittal L_1_(*P* < 0.05) (Table 1). The sagittal and vertical measurement values of the ANB and MP-SN angles, respectively, decreased significantly after treatment(*P* < 0.01), whereas the S-Go/N-Me (%) significantly increased(*P* < 0.01), and the XiPm-SN angle reduced considerably(*P* < 0.01). The U_1_-SN angle and the overjet significantly declined(*P* < 0.01, Table 2). Furthermore, the upper airway sagittal parameters, U-MPW and PAS，were significantly increased after treatment(*P* < 0.05, Table 2 and Fig. 3A). However, after treatment, the hyoid bone position parameters were significantly unchanged (Table 2). The Vt, upper airway segment volume, Hv, Hg, S_1_, S_2_, sagittal L_1_, transverse L_1_, and the IAV did not change significantly. Moreover, the sagittal L_2_ and transverse L_2_ were altered significantly(*P* < 0.01), such that sagittal L_2_ and the transverse L_2_ significantly increased and decreased, respectively (Table 3 and Figs. 2C, D and 3B).

**Table 1:** The differences in 3D airway measurements between T_0_ and the control group

	T_0_	Control group	*t* or *Z*	*P-*values
Vn (mm^3^)	7709.77 ± 2185.89	5498.89/6725.94/9416.39	−0.477	0.633
Vv (mm^3^)	9562.01 ± 3979.64	8108.35/9108.08/13752.94	−0.933	0.351
Vg (mm^3^)	3597.94/5022.80/8238.40	3661.81/6086.10/8282.18	−0.684	0.494
Vl (mm^3^)	1462.19/2549.57/5678.21	4832.917 ± 1876.317	−1.390	0.165
Vt (mm^3^)	26,457.67 ± 9507.23	24,001.90/25574.97/30159.24	−1.224	0.221
Hv (mm)	27.29 ± 4.31	23.68 ± 2.43	3.671	0.003**
Hg (mm)	24.82 ± 2.51	21.61 ± 5.77	1.974	0.058
S_1_ (mm^2^)	220.96 ± 133.11	194.72/234.35/287.08	−1.472	0.141
S_2_ (mm^2^)	219.03 ± 125.65	171.20/206.48/342.42	−1.224	0.221
Sagittal diameter L_1_ (mm)	10.15 ± 3.00	12.81 ± 3.15	−2.347	0.025*
Transverse diameterL_1_ (mm)	25.66 ± 6.15	23.25/24.87/28.21	−0.062	0.950
Sagittal diameter L_2_ (mm)	7.22/12.37/16.51	9.79/11.76/14.43	−1.721	0.085
Transverse diameterL_2_ (mm)	29.39 ± 5.61	25.98 ± 4.23	0.788	0.437
IAV (mm^3^)	23.78/922.02/3197.86	619.18/3373.22/4776.91	−1.597	0.110

*T*_*0*_ Before treatment, *t* Paired *t*-test, *Z* Wilcoxon signed-rank test, *3D* Three-dimensional; *P < 0.05, **P < 0.01

**Table 2:** Changes in the craniomaxillo facial structure, sagittal size of the airway, and hyoid position before and after treatment

	T_0_	T_1_	*t* or *Z*	*P-*values
SNA (°)	80.34 ± 2.51	80.15 ± 2.81	0.521	0.612
SNB (°)	74.75 ± 4.43	75.18 ± 3.41	−0.695	0.504
ANB (°)	4.40/6.02/10.35	4.00/4.56/5.59	−2.612	0.009**
MP-SN (°)	42.85 ± 4.38	41.21 ± 4.21	6.994	0.000**
Y-axis (°)	65.56 ± 3.90	55.20 ± 2.21	−0.643	0.543
ANS-Me/N-Me (%)	55.38 ± 1.97	29.50 ± 4.03	1.054	0.312
S-Go/N-Me(%)	60.84 ± 2.33	61.72 ± 2.46	−5.100	0.000**
XiPm-SN (°)	50.00/53.00/55.00	47.00/50.00/52.00	−2.896	0.004**
U_1_-SN (°)	105.62 ± 9.38	100.37 ± 6.02	3.052	0.009**
L_1_-MP (°)	93.98 ± 6.49	93.13 ± 6.63	0.584	0.565
Overjet (mm)	5.36 ± 2.26	3.50 ± 0.67	3.222	0.006**
Overbite (mm)	0.77/1.52/2.90	1.05/1.63/1.90	−0.463	0.653
PNS-R (mm)	16.6/23.80/27.60	21.10/26.10/28.50	−0.431	0.665
PNS-UPW (mm)	17.40/20.00/22.90	19.60/20.60/23.20	−0.824	0.410
SPP-SPPW (mm)	7.30/9.30/10.90	7.70/9.70/12.40	−1.531	0.133
U-MPW (mm)	4.20/5.10/7.70	5.30/6.90/9.10	−2.392	0.017*
PAS (mm)	6.90/10.50/15.50	8.60/13.50/15.10	−2.292	0.022*
V-LPW (mm)	10.30/11.60/13.60	10.00/11.40/15.10	−0.234	0.821
H-MP (mm)	7.10/10.20/14.60	8.10/10.40/14.70	−0.062	0.963
H-FH (mm)	66.92 ± 10.63	65.47 ± 10.09	1.216	0.242
H-PNS (mm)	44.30/48.80/51.30	44.10/47.00/50.60	−0.573	0.574
H-C3la (mm)	22.10/24.20/26.80	21.90/25.10/27.90	−0.252	0.801
H-NPog (mm)	30.00/34.10/40.00	31.40/36.30/39.40	−0.060	0.957

*T*_*0*_ Before treatment, *T*_*1*_ After treatment, *t* Paired *t*-test, *Z* Wilcoxon signed-rank test; *P < 0.05, **P < 0.01

**Table 3:** Changes in airway size in 3D before and after treatment

	T_0_	T_1_	*t* or *Z*	*P-*values
Vn (mm^3^)	7709.77 ± 2185.89	7739.88 ± 2573.23	−0.093	0.925
Vv (mm^3^)	9562.01 ± 3979.64	10,617.90 ± 4960.36	−0.472	0.653
Vg (mm^3^)	3597.94/5022.80/8238.40	3548.11/4365.34/6551.65	−0.804	0.432
Vl (mm^3^)	1462.19/2549.57/5678.21	2098.42/3769.39/4335.40	−0.062	0.966
Vt (mm^3^)	26,457.67 ± 9507.23	27,928.48 ± 11,172.91	−0.724	0.482
Hv (mm)	27.29 ± 4.31	27.44 ± 4.26	−0.524	0.608
Hg (mm)	24.82 ± 2.51	23.13 ± 3.86	1.983	0.061
S_1_ (mm^2^)	220.96 ± 133.11	215.89 ± 116.36	0.182	0.860
S_2_ (mm^2^)	219.03 ± 125.65	215.96 ± 153.07	0.084	0.942
Sagittal diameterL_1_ (mm)	10.15 ± 3.00	10.27 ± 3.02	−0.187	0.853
Transverse diameterL_1_ (mm)	25.66 ± 6.15	26.35 ± 6.80	−0.472	0.649
Sagittal diameterL_2_ (mm)	7.22/12.37/16.51	9.16/12.27/15.51	−3.124	0.002**
Transverse diameterL_2_ (mm)	29.39 ± 5.61	27.04 ± 5.80	4.028	0.001**
IAV (mm^3^)	23.78/922.02/3197.86	37.16/508.29/4067.53	−1.082	0.282

*T*_*0*_ Before treatment, *T*_*1*_ After treatment, *t* Paired *t*-test, *Z* Wilcoxon signed-rank test, *3D* Three-dimensional; *P < 0.05, **P < 0.01

Furthermore, a Pearson correlation test was performed on the treatment results with significant changes. After treatment, ANB angle, MP-SN angle, S-Go/N-Me (%), XiPm-SN angle, and U-MPW, PAS were not significantly correlated (Table 4). The correlations between the ANB angle and sagittal L_2_, transverse L_2_ were insignificant (Table 4). Conversely, significant correlations were observed between the MP-SN angle, S-Go/N-Me (%) changes, and sagittal L_2_ (*P* < 0.05, Table 4, Fig. 3C, D). In addition, no significant correlations were observed between U-MPW, PAS, sagittal L_2_, transverse L_2_ changes, and U_1_-SN and overjet changes (Table 4).

**Table 4:** Correlation between significant changes in craniofacial structure and upper airway

	U-MPW (mm)		PAS (mm)	Sagittal diameter L_2_ (mm)	Transverse diameter L_2_ (mm)
ANB (°)	*r*	0.317	0.000	− 0.216	− 0.190
	*P*	0.249	0.999	0.439	0.498
MP-SN (°)	*r*	−0.130	− 0.082	− 0.522	− 0.113
	*P*	0.642	0.772	0.033*	0.690
S-Go/N-Me (%)	*r*	0.063	− 0.073	0.549	0.004
	*P*	0.824	0.796	0.034*	0.989
XiPm-SN (°)	*r*	− 0.030	− 0.383	− 0.238	0.120
	*P*	0.915	0.158	0.393	0.670
U_1_-SN (°)	*r*	0.101	−0.033	− 0.494	− 0.190
	*P*	0.720	0.907	0.061	0.498
overjet (mm)	*r*	0.389	0.207	−0.315	−0.184
	*P*	0.151	0.459	0.253	0.511

*r* Pearson correlation coefficient, * *P* < 0.05, ** *P* < 0.01, *2D* Two-dimensional, *3D* Three-dimensional, *P < 0.05, **P < 0.01

The Univariate linear regression analysis was performed before performing the Multiple linear regression analysis (Additional file 1). It was used to determine the independent variables. After the Univariate linear regression analysis, we found that the variables ANB and ANS-Me/N-Me (%) were negatively correlated with the variables S_1_ and PNS-UPW respectively(*P* < 0.05), the variable U_1_-SN was negatively correlated with the variable IAV but positively correlated with sagittal L_2_ (*P* < 0.05), the variable overjet was negatively correlated with transverse L_1_ but positively correlated with S_2_(*P* < 0.05), the variable overbite was positively correlated with PNS-UPW and U-MPW(*P* < 0.05). However, the variables MP-SN and S-Go/N-Me(%) were negatively and positively correlated with sagittal L_2_ (*P* < 0.01), PNS-UPW(*P* < 0.05) and H-MP(*P* < 0.05) variables, respectively. Therefore, there were more relevant variables related to MP-SN and S-Go/N-Me(%) and they were all related to upper airway and hyoid position variables. We chose these two variables as independent variables. The Multiple linear regression analysis showed that there was a significant negative correlation between the variables MP-SN and sagittal diameter L_2_ and positive correlation between S-Go/N-Me(%) and H-MP(*P* < 0.05) (Table 5).

**Table 5:** Correlation analysis between independent variables and dependent variables tested by multivariable linear regression analysis

Dependent variables	Independent variables			
	Mp-SN(°)		S-Go/N-Me(%)	
	β	*P-values*	β	*P-values*
Vn (mm^3^)	−0.212	0.602	0.004	0.993
Vv (mm^3^)	−0.359	0.375	−0.161	0.686
V_g_ (mm^3^)	−0.158	0.280	0.280	0.759
Vl (mm^3^)	−0.232	0.563	−0.375	0.355
Vt (mm^3^)	0.274	0.502	−0.125	0.757
Hv (mm)	0.716	0.057	0.713	0.052
Hg (mm)	−0.005	0.991	−0.005	0.991
S_1_ (mm^2^)	0.214	0.543	0.004	0.991
S_2_ (mm^2^)	−0.304	0.439	0.052	0.894
Sagittal diameterL_1_ (mm)	0.051	0.901	0.210	0.607
Sagittal diameterL_2_ (mm)	−0.728	0.041*	0.719	0.061
Transverse diameterL_1_ (mm)	−0.516	0.194	−0.439	0.265
Transverse diameterL_2_ (mm)	−0.453	0.229	−0.660	0.089
IAV (mm^3^)	0.497	0.183	0.006	0.986
PNS-R (mm)	−0.146	0.720	0.059	0.884
PNS-UPW (mm)	−0.327	0.421	−0.022	0.955
SPP-SPPW (mm)	−0.070	0.864	−0.008	0.985
U-MPW (mm)	−0.170	0.680	−0.056	0.891
PAS (mm)	−0.262	0.522	−0.257	0.530
V-LPW (mm)	−0.116	0.779	−0.144	0.359
H-MP (mm)	−0.290	0.467	0.825	0.036*
H-FH (mm)	−0.026	0.949	0.139	0.735
H-PNS (mm)	−0.195	0.631	0.030	0.941
H-C3la (mm)	−0.202	0.622	−0.243	0.553
H-Npog (mm)	−0.167	0.684	−0.064	0.876

The Multiple linear regression analysis was performed using the variables of MP-SN (°) and S-Go/N-Me (%) as the independent variables. *P < 0.05, β: regression coefficient

After treatment, the differences between the experimental and control groups were investigated with no significant differences observed in the volume of each part of the upper airway, Vt, Hg, S_1_, transverse L_1_, sagittal L_2_, transverse L_2_, or IAV. However, significant differences were recorded in the Hv (*P* < 0.01) and sagittal L_1_ and S_2_ *(P* < 0.05) (Table 6).

**Table 6:** The differences in 3D airway measurements between T_1_ and control group

	T_1_	Control group	*t* or *Z*	*P-*values
Vn (mm^3^)	7739.88 ± 2573.23	5498.89/6725.94/9416.39	−0.270	0.787
Vv (mm^3^)	10,617.90 ± 4960.36	8108.35/9108.08/13752.94	−0.436	0.663
Vg (mm^3^)	3548.11/4365.34/6551.65	3661.81/6086.10/8282.18	−1.058	0.290
Vl (mm^3^)	2098.42/3769.39/4335.40	4832.917 ± 1876.317	−2.002	0.055
Vt (mm^3^)	27,928.48 ± 11,172.91	24,001.90/25574.97/30159.24	−1.099	0.272
Hv (mm)	27.44 ± 4.26	23.68 ± 2.43	3.629	0.003**
Hg (mm)	23.13 ± 3.86	21.61 ± 5.77	0.849	0.403
S_1_ (mm^2^)	215.89 ± 116.36	194.72/234.35/287.08	−1.846	0.065
S_2_ (mm^2^)	215.96 ± 153.07	171.20/206.48/342.42	−1.970	0.049*
Sagittal diameterL_1_ (mm)	10.27 ± 3.02	12.81 ± 3.15	−2.252	0.032*
Transverse diameterL_1_ (mm)	26.35 ± 6.80	23.25/24.87/28.21	−0.187	0.870
Sagittal diameterL_2_ (mm)	9.16/12.27/15.51	9.79/11.76/14.43	−0.228	0.820
Transverse diameterL_2_ (mm)	27.04 ± 5.80	25.98 ± 4.23	0.751	0.459
IAV (mm^3^)	37.16/508.29/4067.53	619.18/3373.22/4776.91	−1.929	0.054

*T*_*0*_ Before treatment, *T*_*1*_ After treatment, *t* Paired *t*-test, *Z* Wilcoxon signed-rank test, *3D* Three-dimensional, *P < 0.05, **P < 0.01

## Discussion

CBCT plays a vital role in diagnosing oral and maxillofacial morphological anomalies. Interestingly, it has the advantages of low radiation, low cost, easy acquisition, short scanning time, and accurate delineation of the cavity structure boundaries [8]. Schendel et al. [9] demonstrated that the 3D upper airway volume measurements obtained from CBCT images could accurately signify the anatomical structure and spatial size. However, airway measurements on lateral cranial radiographs can only provide 2D measurements but cannot evaluate the upper airway volume and minimum cross-sectional area, which poses certain limitations.

Jadhav et al. [10] reported that the width of the upper and lower airways in patients with hyperdivergent skeletal Class II was significantly smaller than that in those with hyperdivergent skeletal Class I. Moreover, Oz et al. [11] found that the oropharyngeal segment size in patients with hyperdivergent skeletal Class II group was significantly smaller than that of the normal angle skeletal Class I group. Among the three vertical skeletal Class II groups, the size of the upper airway oropharyngeal segment in the high-angle group was significantly reduced compared to that in the low-and normal-angle groups. Furthermore, Mao et al. [12] showed that the upper airway of patients with hyperdivergent skeletal Class II is narrower and longer than that of those with hyperdivergent skeletal Class I. The study’s result also showed that the mandible length positively correlates with the sagittal and coronal diameters of the velopharyngeal segment, the insufficient mandible length is more likely to affect the velopharyngeal airway morphology. Our study showed that Hv and sagittal L_1_ were significantly different between the skeletal Class II high-angle and the control groups, before treatment, which indicates that the velopharyngeal segment of the patients with skeletal Class II was narrower and longer than that of those with the hyperdivergent skeletal Class I. Notably, the results were similar to those of the above mentioned studies. Therefore, we hypothesize that the reasons for the narrow and long velopharyngeal segment airway are majorly associated with two aspects. First, the pressure changes in the airway lumen when the mandible is rotated posteriorly and inferiorly. This limited change is related to passive compression and stretching of the pharyngeal wall, which may protect against severe airway obstruction. Second, it is associated with the insufficient width of the upper and lower jaws and the posterior segment of the dental arch in patients with hyperdivergent skeletal class II. A possible cause could be that the width is inadequate to limit the inherent oral volume to prevent the tongue from extending forward, and the tongue falls back; therefore, the back of the tongue causes the velopharyngeal segment to be narrowed and elongated. Deng et al. [13] believed that the sagittal and vertical differences of bone in skeletal class II high angle patients were related to the increased risk of OSA. We should focus on the effect of orthodontic camouflage treatment on the upper airway, tongue body and hyoid bone position of these patients to reduce the risk of OSA. To some extent, tooth extraction can compensate for maxillary and mandibular skeletal disorders in adult patients with hyperdivergent skeletal class II. However, due to the particularity of the upper airway in these patients, it is necessary to pay close attention to the changes in the treatment process to avoid the occurrence of iatrogenic upper airway stenosis. At present, there are still controversies on whether the size of the upper airway and position of hyoid bone changes after orthodontic camouflage treatment for patients with hyperdivergent skeletal class II, there are also few studies on whether the change of mandibular position caused by orthodontic camouflage treatment affects the size and morphology of the upper airway. Therefore, this study selected a designated sample and observed whether there was any change in the upper airway after vertical control of orthodontic treatment to provide guidance for clinical work.

Kim et al. [14] found that after treating patients with hyperdivergent skeletal Class II with implant anchorage, for every 1 mm maxillary molar intrusion, the mandibular plane rotated counterclockwise by 2°, and the chin was moved forward by 2.3 mm. Koyama et al. [15] employed implant anchorage to treat patients with a high angle and discovered that the mandibular plane angle reduced by an average of 1.5° while the maxillary molar height decreased by 0.7 mm after treatment. In this study, implant anchorage was employed to actively intrude the bilateral maxillary posterior segments and upright mandibular molars and move them mesially as a whole. Consequently, the occlusal plane remained flat, the mandibular plane had a certain degree of counterclockwise rotation, the MP-SN angle decreased by an average of 1.64 ± 0.17° (*P* < 0.01), and the patient’s profile was also significantly improved.

The sagittal U-MPW and PAS of the upper airway near the mandible were significantly altered after treatment, which is consistent with Germec-Cakan et al. [16], who suggested the increase in the sagittal size of the upper airway near the mandible was due to the mesial movement of the molars. Hyoid bone is a unique part of the axial bone. It is connected with the pharynx, skull and mandible through muscles and ligaments to form the oropharyngeal complex [17]. Although the final result of the Multiple linear regression analysis showed that when S-Go/N-Me(%) increased, H-MP also increased accordingly, there was no significant change in the hyoid bone position after treatment was observed, which was consistent with Shi et al. [6] and Zhang et al. [4]. Moreover, LaBanc et al. [18] supposed that the change in the mandibular position would stretch the muscles and tendons attached to the hyoid bone, increase the anterior abdominal region of the digastric muscle, and eventually return the hyoid bone to the pre-treatment position. Therefore, the hyoid bone position did not change significantly, which may be attributed to the instability in the adaptive remodeling of the muscle and its rebound and pulling effect. Compared to the patients with hyperdivergent skeletal Class I, the tongue position is low in those with hyperdivergent skeletal Class II, and the tongue body is small; the backward drop of the tongue can lead to a narrower upper airway [19]. In this study, the size of IAV was measured to research whether the position of tongue changed. Our study’s results showed that the tongue body position did not change significantly after treatment, possibly due to the limited significant alterations in the hyoid bone position. Moreover, Aras et al. [20] reported that the mandible’s forward motion did not significantly change the tongue body and hyoid bone positions and believed that the tongue posture was associated with the hyoid bone position. Santos et al. [21] reported that the hyoid bone can play a role in fixing the hyoid muscle and that its position affects the tongue body’s position, size, and shape. The volume of the upper airway segments and total volume did not change significantly after the treatment, which aligned with the findings of previous studies [4, 6, 22–24] that proposed a negligible effect of orthodontic treatment on the size of the upper airway space in adults. However, no significant changes occurred in Hv, Hg, S_1_, S_2_, sagittal L_1_, and transverse L_1_, contrary to the findings of Hu et al. [25] and Sun et al. [26]. They believe that large upper anterior teeth are retracted to cause the tongue to move backward, compress the soft palate, and reduce S_1_ and S_2_. Conversely, this study’s results showed that the sagittal L_2_ and transverse L_2_ were significantly increased and reduced, respectively, although the minimum cross-sectional area of the glossopharyngeal segment did not change significantly. During the treatment, the upper airway was only self-regulated to permit ventilation, with no actual size change. This finding is consistent with that of Zhang et al. [4], who believed that the minimum cross-sectional area of the oropharyngeal segment remained stable in adults with hyperdivergent skeletal Class II who received strong anchorage retraction of the upper anterior teeth with the assistance of MIA. In addition, the volume of each upper airway segment did not change significantly. Therefore, it is speculated that the upper airway only undergoes adaptive changes after treatment.

Furthermore, no significant correlation was observed between the ANB angle of the sagittal change of the jaw and that in U-MPW and PAS, which corresponds with the findings of Chokotiya et al. [27]. Moreover, a significant correlation existed between the vertical changes in the jaws’ MP-SN angle, S-Go/N-Me (%), and that in the sagittal L_2_ rather than that in the transverse L_2_. Meanwhile, the Multiple linear regression analysis showed that there was a significant negative correlation between the variables MP-SN and sagittal L_2,_ but there was no significant correlation with transverse L_2_. All these indicated that the mandibular position change did not cause a change in S_2_. However, this result was inconsistent with that of Shi et al. [6], who reported that an increase in the minimum cross-sectional area of the upper airway was significantly linked to changes in the mandibular position. Additionally, the result showed that U-MPW, PAS, sagittal L_2_, and transverse L_2_ changes were not connected to the significant changes in the dentition, U_1_-SN, and overjet, which is consistent with Valiathan et al. [23], and contrary to Chen et al. [28], who found that the extraction of four premolars and massive retraction of anterior teeth reduced the S_2_ and considered that it was majorly attributed to the reduction of oral volume caused by retraction of the upper anterior teeth, which affected the tongue position. However, no significant changes in tongue position were found in this study, but only the self-regulation of glossopharynx occurred, without actual size changes. The Hv and sagittal L_1_ were significantly different from the individual normal groups, indicating that even after treatment, the velopharyngeal segment of patients with skeletal Class II high angle was narrower and longer than that of the control group, which was shown by the differential analysis of the 3D direction measurement indices between the experimental and control groups. However, this was considered due to the tongue body position, which did not change significantly after treatment, but still fell. Compared with the control group, the S_2_ showed a significant difference after treatment (*p* = 0.049, *p* < 0.05), but the S_2_ of the experimental group and the control group did not show a significant difference before treatment, and the sagittal diameter L_2_ increased before and after treatment, the transverse diameter L_2_ decreased, and S_2_ did not change significantly, which was considered to be related to the self-regulation and adaptive changes of the glossopharyngeal airway.

This study had some limitations. First, this was a single-center study with small sample size. Therefore, a multi-center study should be conducted in future studies, and the sample size should be increased. Additionally, during orthodontic camouflage treatment of adult patients with hyperdivergent skeletal class II, in order to maintain oral and maxillofacial health, stomatologists should focus on improving the shape of the upper airway and avoiding iatrogenic upper airway stenosis. In future research, performing a joint multidisciplinary survey with ENT and other disciplines is crucial to verify the size and changes in the relationship between the upper respiratory tract and respiratory function.

## Conclusions

Adult patients with hyperdivergent skeletal Class II high-angle had narrower and longer velopharyngeal airways than the individual normal patients before orthodontic treatment. However, the velopharyngeal airway was still narrow and long after the orthodontic vertical control treatment. Additionally, the sagittal dimension in the 2D direction near the mandible increased significantly, and the airway height, minimum cross-sectional area, volume in the 3D direction, tongue body, and hyoid bone did not change considerably after the orthodontic vertical control treatment. Furthermore, only the sagittal L_2_ was significantly increased, while the transverse L_2_ decreased significantly. Moreover, the S_2_ remained unchanged, and the upper airway only achieved self-regulation and adaptive changes during the treatment process.

## Supplementary Information

**Additional file 1.** The Univariate linear regression analysis of the relationship between the variables of craniomaxillofacial structure and the size of upper airway and the position of tongue and hyoid bone; Description of data: See the results section for details.

## Data Availability

All data generated or analyzed during this study are included in this published article.

## Abbreviations

CBCT: Cone beam Computer Tomography
3D: The three-dimensional
2D: The two-dimensional
MIA: Micro-implant anchorage
MBT: Straight arch-wire technology
